# Predictive factors for effectiveness and safety of enoxaparin for total knee arthroplasty in aged Japanese patients: a retrospective review

**DOI:** 10.1186/s40780-017-0075-x

**Published:** 2017-01-18

**Authors:** Akihiro Sonoda, Yuki Kondo, Yasuhiro Tsuneyoshi, Yoshitaka Iwashita, Shoji Nakao, Kazuhisa Ishida, Kentaro Oniki, Junji Saruwatari, Tetsumi Irie, Yoichi Ishitsuka

**Affiliations:** 1 Department of Clinical Chemistry and Informatics, Graduate School of Pharmaceutical Sciences, Kumamoto University, 5-1 Oe-honmachi, Kumamoto, Chuo-ku 862-0973 Japan; 2 Department of Pharmacy, Izumi Regional Medical Center, 4513 Akasegawa, Akune, Kagoshima 899-1611 Japan; 3 Department of Orthopedic Surgery, Izumi Regional Medical Center, 4513 Akasegawa, Akune, Kagoshima 899-1611 Japan; 4 Division of Pharmacology and Therapeutics, Graduate School of Pharmaceutical Sciences, Kumamoto University, 5-1 Oe-honmachi, Kumamoto, Chuo-ku 862-0973 Japan; 5 Center for Clinical Pharmaceutical Sciences, Faculty of Pharmaceutical Sciences, Kumamoto University, 5-1 Oe-honmachi, Kumamoto, Chuo-ku 862-0973 Japan

**Keywords:** Enoxaparin, Total knee arthroplasty, Adverse drug event, Serum total protein, Anemia, Venous thromboembolism

## Abstract

**Background:**

The aims of this study were to investigate predictive factors involved in effectiveness and safety of enoxaparin for prevention of postoperative venous thromboembolism in aged Japanese total knee arthroplasty (TKA) patients.

**Methods:**

Japanese patients over 65 years old who were administered enoxaparin for TKA were enrolled in this study. Their medical records were retrospectively reviewed. Data were corrected at the Izumi Regional Medical Center, from September 2009 to March 2014. Patients were stratified into groups according to whether enoxaparin was effective (no deep vein thrombosis event up to postoperative day 7) or not, and whether they had an adverse drug event (ADE) or not.

**Results:**

A total of 128 patients were included in this study. One hundred five (82.0%) patients were in the effective group and 20 (15.6%) in the adverse drug event (ADE) group. Anemia (13 patients), abnormalities in liver function tests (4 patients), clinically relevant non-major bleeding (4 patients) and urticaria (1 patient) were observed as ADEs. Multivariate logistic regression analysis showed that the serum total protein level at postoperative day 1 (POD1, before enoxaparin administration), was associated with effectiveness of enoxaparin, while the serum total protein and hemoglobin level at POD1 were involved in ADE caused by enoxaparin.

**Conclusions:**

Although further large scale studies will be warranted, our results suggest that serum total protein level just before enoxaparin treatment for TKA relates to the effectiveness and safety of enoxaparin in a Japanese aged population. In addition, the results indicate that the development of anemia should be carefully monitored during enoxaparin treatment for TKA, particularly in patients with lower levels of serum hemoglobin before treatment.

**Electronic supplementary material:**

The online version of this article (doi:10.1186/s40780-017-0075-x) contains supplementary material, which is available to authorized users.

## Background

Venous thromboembolism (VTE) is common after total knee arthroplasty (TKA), and its incidence is particularly high after orthopedic surgery. For example, the incidence of deep vein thrombosis (DVT) and pulmonary embolism after knee arthroplasty are 41–85% and 1.5–10%, respectively, without anticoagulant therapy [1].

Enoxaparin sodium (enoxaparin), a low-molecular-weight heparin, has been used to prevent VTE after TKA, and has been shown to be effective in clinical trials [2, 3]. Generally, enoxaparin has a predictable pharmacokinetic profile and dose response curve, allowing simplified dosing without the need for careful monitoring through laboratory tests [4]. However, the standard dose of enoxaparin is sometimes ineffective in DVT high risk groups, such as intensive care patients [5]. In addition, enoxaparin has been associated with severe adverse drug events (ADE), such as bleeding, skin reaction, liver failure, and anemia [6–8]. ncreasing age is a common risk factor for ADE with many types of medication [9–11], and enoxaparin is no exception. Macie et al. suggested that increasing age was a risk factor for bleeding during treatment with enoxaparin [12]. Additionally, patient age was related to the efficacy of enoxaparin in Phase III clinical trials (Trial number: EFC10094, PK568, and PK567). However, it is unclear which factors are associated with the effectiveness and/or ADE of enoxaparin in aged patients.

Finding the predictive factors involved in the effectiveness and/or ADE of anticoagulant therapy in aged patients is critical to avoid severe DVT and/or ADE. We conducted this study to investigate the predictive factors involved in the effectiveness and/or safety of enoxaparin for TKA in aged Japanese patients.

## Methods

### Study design

We retrospectively analyzed the medical records of all patients aged 65 years or older who were administered enoxaparin for TKA. Data were corrected at the Izumi Regional Medical Center, from September 2009 to March 2014. No exclusion criteria were set.

The following data were collected: age, sex, height, body weight, enoxaparin dose, previous history of DVT, use of tourniquet, operative time, volume of bleeding during surgery, laboratory values at postoperative day 1 (POD1) as the laboratory values before enoxaparin administration, complications (hypertension, diabetes, dyslipidemia, chronic heart failure, cerebral vascular disease), concomitant medications that may interact with enoxaparin (non-steroidal anti-inflammatory drugs, low dose aspirin, clopidogrel, ticlopidine, cilostazol, limaprost alfadex, warfarin, raloxifene).

To evaluate effectiveness, the patients were classified into two groups, the “effective” group and “ineffective” group. In this study, “effective” was defined as no DVT event up to postoperative day 7. The safety of enoxaparin was evaluated according to presence (ADE (+) group) or absence (ADE (−) group) of ADE. The laboratory values at POD7 were used for the assessment of laboratory abnormalities. The ADE in this study were defined as > grade 3 according to the National Cancer Institute Common Terminology Criteria for Adverse Events (version 4.0) [13] and/or clinically relevant non-major (CRNM) bleeding [14]. This study was approved by the Ethics Committee of Kumamoto University (no. 915) and Izumi Regional Medical Center (no. 20140908–1). All analyses were conducted using anonymized data.

### Statistical analysis

The statistical power of the association analyses of the effectiveness and ADE of enoxaparin were calculated at a significance (alpha) level of 0.05 (two-tailed) and an effect size (zeta) of 0.2–1.0 according to the sample size of the present study using the G*Power software program (version 3.1.9.2).

Continuous variables are expressed as mean ± standard deviation or median (range). The normality of data were assessed using Shapiro-Wilk test. Univariate analyses to compare two groups were performed using Welch’s *t* test, Mann–Whitney *U* test or Fisher's exact test. Multivariate logistic regression analysis was used to test the outcomes from univariate analysis. Parameters that showed a correlation (*P* < 0.2) in the univariate analysis were included in the multivariate analysis. Logistic regression was performed using stepwise model selection according to Bayesian information criterion [15]. A receiver operating characteristic (ROC) curve for the significantly affecting factor of effectiveness and safety of enoxaparin was plotted. Additionally, the cut-off values were decided by Youden index. Significance values were set at *P* < 0.05 for interpretation of the final multivariate logistic regression model. The statistical analyses were performed with JMP® Pro 12 (SAS Institute Inc., Cary, NC, USA).

## Results

### Effectiveness of enoxaparin

A total of 128 patients were included in this study. One hundred five (82.0%) patients were classified as the effective group. The characteristics of the effective and ineffective groups are shown in Table 1. In univariate analysis, serum total protein level at POD1, and the number of patients who used a tourniquet were significantly different between two groups. We evaluated the incidence rate of ADE in the efficacy and ineffective groups, and no significant difference was observed between the groups (Additional file 1: Table S1).

**Table 1:** Characteristics of patients classified according to effectiveness of enoxaparin

	Effective	Ineffective	*P* value
Case, no. (%)	105 (82.0)	23 (18.0)	-
Age (years)^a^	77.2 ± 5.11	79.0 ± 4.51	0.10
Sex, no. (%)			
male	25 (23.8)	3 (13.0)	0.40
female	80 (76.2)	20 (87.0)	
Height (cm)^b^	150 (135–176)	145 (140–165)	0.08
Body weight (kg)^b^	57 (38–91)	51 (39–69)	0.11
Enoxaparin dose, no. (%)			
2000 mg/day	36 (34.3)	9 (39.1)	0.64
4000 mg/day	69 (65.7)	14 (60.9)	
Previous history of DVT, no. (%)			
yes	4 (3.81)	3 (13.0)	0.11
Use of tourniquet, no. (%)			
yes	35 (33.3)	15 (65.2)	<0.01
Operative time, (min)^b^	110 (65–260)	100 (75–210)	0.14
Volume of bleeding during surgery (mL)^b^	100 (0–1530)	70 (0–500)	0.33
Laboratory values at POD1			
Total serum protein (g/dL)^a^	5.85 ± 0.60	6.35 ± 0.53	<0.001
Serum creatinine (mg/dL)^b^	0.68 (0.35–2.13)	0.64 (0.43–1.07)	0.15
BUN (mg/dL)^b^	13.5 (7.4–24.3)	12.0 (8.6–20.0)	0.16
AST (IU/L)^b^	20.0 (11.0–80.0)	20.0 (11.0–44.0)	0.68
ALT (IU/L)^b^	13.0 (5.0–50.0)	12.0 (6.0–57.0)	0.50
LDH (IU/L)^b^	199 (122–390)	216 (134–273)	0.20
ALP (IU/L)^b^	185 (94–398)	181 (122–337)	0.78
γ-GTP (IU/L)^b^	14 (7–460)	13 (8–143)	0.69
ChE (IU/L)^b^	220 (108–401)	231 (144–316)	0.90
Total bilirubin (mg/dL)^b^	0.8 (0.3–2.7)	0.9 (0.4–1.3)	0.74
WBC (10^3^/μL)^b^	90 (45–405)	82 (62–158)	0.57
Hb (g/dL)^a^	10.9 ± 1.27	10.6 ± 1.21	0.28
Platelet (10^4^/μL)^a^	18.2 ± 5.93	17.7 ± 4.61	0.62
CRP (mg/dL)^b^	4.57 (1.58–12)	5.52 (1.04–14.04)	0.41
Complications, no. (%)			
Hypertension	72 (68.6)	19 (82.6)	0.21
Diabetes	13 (12.4)	2 (8.70)	1.00
Dyslipidemia	30 (28.6)	6 (26.1)	1.00
Chronic heart failure	6 (5.71)	0 (0.00)	0.59
Cerebral vascular disease	6 (5.71)	0 (0.00)	0.59
Concomitant drugs, no. (%)			
NSAIDs	100 (95.2)	21 (91.3)	0.61
Low dose aspirin	12 (11.4)	2 (8.70)	1.00
Clopidogrel	2 (1.90)	1 (4.35)	0.45
Ticlopidine	1 (0.95)	1 (4.35)	0.33
Cilostazol	1 (0.95)	1 (4.35)	0.33
Limaprost alfadex	5 (4.76)	3 (13.0)	0.15
Warfarin	6 (5.71)	1 (4.35)	1.00
Raloxifene	4 (3.81)	2 (8.70)	0.29

^a^Data are expressed as mean ± SD, ^b^Data are expressed as median (range)

*SE* indicates standard error, *POD1* post-operative day 1, *BUN* blood urea nitrogen, *AST* aspartate aminotransferase, *ALT* alanine aminotransferase, *LDH* lactate dehydrogenase, *ALP* alkaline phosphatase, γ*-GTP* γ-glutamyl transferase, *ChE* serum cholinesterase, *WBC* white blood cells, *Hb* hemoglobin, *CRP* C-reactive protein, *NSAIDs* non-steroid anti-inflammatory drugs

**Fig. 1:**
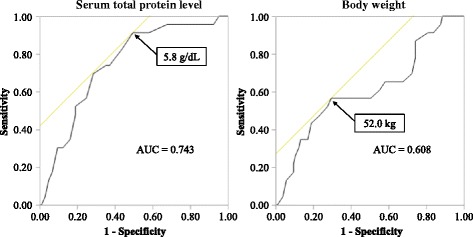
Receiver operating characteristic curve for predicting effectiveness of enoxaparin with serum total protein at POD 1. *POD 1* indicates post-operative day 1, *AUC* area under the curve

The final model of multivariate logistic regression is shown in Table 2. The serum total protein level at POD1 and body weight were identified as predictive factors of effectiveness of enoxaparin (odds ratio: 0.22 and 1.06, respectively). The ROC curve of serum protein level at POD1 and body weight are shown in Fig. 1. The cut-off value of serum protein level at POD1 and body weight were 5.8 g/dL (specificity: 50.5%, sensitivity: 91.3%) and 52.0 kg (specificity: 70.5%, sensitivity: 56.5%), respectively (Fig. 1).

**Table 2:** Multivariate logistic regression analysis of the factors associated with effective of enoxaparin

	Odds ratio (95% CI)	*P* value
Serum total protein at POD1	0.22 (0.10–0.52)	0.0002
Body weight	1.06 (1.00–1.11)	0.0239

*POD1* indicates post-operative day 1, *CI* confidence interval

### Safety of enoxaparin

Twenty (15.6%) patients were classified as the ADE (+) group. The characteristics of the ADE (+) and ADE (−) groups are shown in Table 3. Of the patients in the ADE (+) group, 13 had anemia, 4 had elevation of liver function tests, 4 had CRNM bleeding and 1 had urticaria (see Additional file 2: Table S2). The serum total protein level, lactate dehydrogenase level, and hemoglobin level at POD1 were significantly lower in the ADE (+) group than the ADE (−) group.

**Table 3:** Characteristics of patients classified according to presence or absence of severe adverse drug events after enoxaparin administration

	ADE (−)	ADE (+)	*P* value
Case, no. (%)	108 (84.4)	20 (15.6)	-
Age (years)^a^	77.7 ± 5.00	76.5 ± 5.26	0.34
Sex, no. (%)			
male	23 (21.3)	5 (25.0)	0.77
female	85 (78.7)	15 (75.0)	
Height (cm)^b^	150 (138–175)	146 (135–176)	0.67
Body weight (kg)^b^	58 (38–85)	53.5 (39–91)	0.31
Enoxaparin dose, no. (%)			
2000 mg/day	41 (38.0)	4 (20.0)	0.14
4000 mg/day	67 (62.0)	16 (80.0)	
Previous history of DVT, no. (%)			
yes	5 (4.63)	2 (10.0)	0.30
Use of tourniquet, no. (%)			
yes	42 (38.9)	8 (40.0)	1.00
Operative time, (min)^b^	107 (65–260)	117.5 (85–230)	0.03
Volume of bleeding during surgery (mL)^b^	90 (0–1530)	115 (0–420)	0.83
Laboratory values at POD1			
Total serum protein (g/dL)^a^	6.03 ± 0.59	5.46 ± 0.57	<0.01
Serum creatinine (mg/dL)^b^	0.67 (0.35–2.13)	0.67 (0.46–1.08)	0.88
BUN (mg/dL)^b^	12.9 (7.4–23.3)	12.65 (9.2–24.3)	0.76
AST (IU/L)^b^	20.0 (11.0–52.0)	18.5 (11.0–80.0)	0.25
ALT (IU/L)^b^	13.0 (5.0–57.0)	12.0 (6.0–50.0)	0.39
LDH (IU/L)^b^	204.5 (131–390)	172 (122–250)	<0.01
ALP (IU/L)^b^	185 (94–337)	188 (107–398)	0.86
γ-GTP (IU/L)^b^	14 (8–143)	11.5 (7–460)	0.23
ChE (IU/L)^b^	229 (128–392)	205 (108–401)	0.09
Total bilirubin (mg/dL)^b^	0.9 (0.3–2.7)	0.8 (0.4–2.0)	0.18
WBC (10^3^/μL)^b^	85.5 (45–405)	94 (58–140)	0.35
Hb (g/dL)^a^	11.0 ± 1.17	9.75 ± 1.21	<0.01
Platelet (10^4^/μL)^a^	18.3 ± 5.52	17.3 ± 6.75	0.56
CRP (mg/dL)^b^	4.56 (1.04–14.04)	5.425 (2.29–8.42)	0.20
Complications, no. (%)			
Hypertension	79 (73.2)	12 (60.0)	0.28
Diabetes	14 (13.0)	1 (5.0)	0.46
Dyslipidemia	33 (30.6)	3 (15.0)	0.19
Chronic heart failure	4 (3.70)	2 (10.0)	0.24
Cerebral vascular disease	5 (4.63)	1 (5.0)	1.00
Concomitant drugs, no. (%)			
NSAIDs	102 (94.4)	19 (95.0)	1.00
Low dose aspirin	13 (12.0)	1 (5.0)	0.70
Clopidogrel	3 (2.78)	0 (0.0)	1.00
Ticlopidine	2 (1.85)	0 (0.0)	1.00
Cilostazol	2 (1.85)	0 (0.0)	1.00
Limaprost alfadex	7 (6.48)	1 (5.0)	1.00
Warfarin	7 (6.48)	0 (0.0)	0.60
Raloxifene	5 (4.63)	1 (5.0)	1.00

^a^Data are expressed as mean ± SD, ^b^Data are expressed as median (range)

*ADE (−)* indicates absence of adverse drug event*s, ADE (+)* presence of adverse drug events, *SE* standard error, *POD1* post-operative day 1, *BUN* blood urea nitrogen, *AST* aspartate aminotransferase, *ALT* alanine aminotransferase, *LDH* lactate dehydrogenase, *ALP* alkaline phosphatase, γ*-GTP* γ-glutamyl transferase, *ChE* serum cholinesterase, *WBC* white blood cells, *Hb* hemoglobin, *CRP* C-reactive protein, *NSAIDs* non-steroid anti-inflammatory drugs

**Fig. 2:**
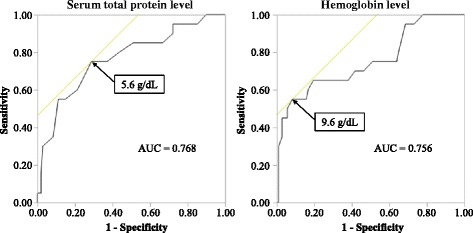
Receiver operating characteristic curve for predicting adverse drug events with enoxaparin with total protein level and hemoglobin level at POD 1. *POD 1* indicates post-operative day 1, *AUC* area under the curve

In multivariate logistic regression, the serum total protein level and hemoglobin level at POD1 were associated with ADE caused by enoxaparin (Table 4). The ROC curves of serum total protein level and hemoglobin level at POD1 are shown in Fig 2. The cut-off value of serum total protein level and hemoglobin level at POD1 were 5.6 g/dL (specificity: 71.3%, sensitivity: 75.0%) and 9.6 g/dL (specificity: 91.7%, sensitivity: 55.0%), respectively.

**Table 4:** Multivariate logistic regression analysis of the factors associated with adverse drug events with enoxaparin

	Odds ratio (95% CI)	*P* value
Serum protein at POD1	0.27 (0.08–0.78)	0.0142
Hemoglobin	0.44 (0.24–0.75)	0.0022

*POD 1* indicates post-operative day 1, *CI* confidence interval

### Statistical powers

The statistical powers of the association analyses of the effectiveness and ADE of enoxaparin with the factors were 31.4 to 92.4% and 30.3 to 90.5%, respectively.

## Discussion

In our study, we demonstrated that the predictive factor for effectiveness of enoxaparin for TKA in aged Japanese patients was serum protein level at POD1. We also demonstrated that the serum total protein level and hemoglobin level at POD1 were associated with ADE of enoxaparin.

DVT is associated with morbidity and mortality in hospitalized patients [16–18]. Hull et al. [19] reported that rapidly achieving therapeutic levels of heparin can improve prognosis of DVT. Therefore, the prediction of enoxaparin effectiveness is clinically important to detect DVT early. Our results indicated that the serum total protein level at POD1 was lower in the effective group than the ineffective group, and body weight was higher in the effective group than the ineffective group. (Tables 1 and 2), and the cut-off value of serum total protein level at POD1 was 5.8 g/dL (Fig. 1). The AUC of serum total protein level at POD1 was classified as having moderate accuracy (0.7 < AUC ≤ 0.9) [20], while the AUC of body weight was classified as having low accuracy (0.5 < AUC ≤ 0.7). These findings suggest that the serum total protein level prior to administration of enoxaparin is a useful predictive factor to improve prognosis of DVT.

Our data demonstrated that the serum total protein level at POD1 was lower in the ADE (+) than ADE (−) groups (Tables 3 and 4), and the cut-off value was 5.6 g/dL (Fig. 2). These results show that the serum total protein level prior to administration of enoxaparin may also be associated with incidence of ADE caused by enoxaparin.

In general, the pharmacokinetics and pharmacodynamics of highly protein-bound drugs are affected by plasma protein concentrations [21–23]. For example, hypoalbuminemia was associated with an increased risk of over-anticoagulation by warfarin [24]. Enoxaparin is a highly plasma protein-bound drug (Clexane® [interview form]. KAKEN PHARMACEUTICAL CO.,LTD., Japan; Mar 2015, http://www.kaken.co.jp/medical/if/clexane_201503if.pdf, Accessed Dec 20, 2016). Based on this fact and our findings, it appears that the pharmacology and ADE of enoxaparin might be enhanced by hypoproteinemia. Additionally, hypoalbuminemia is a major complication after surgery, including TKA [25–27]. Kumar et al. [28] reported that hypoproteinemia is a risk factor for DVT. These findings suggest that hypoproteinemia is indirectly associated with the efficacy of enoxaparin. Taken together, our results suggest that monitoring of total serum protein level is critical to avoid DVT and ADE in TKA patients.

In this study, the hemoglobin level at POD1 was also associated with ADE caused by enoxaparin (Table 4). Anemia was the most common ADE in our study and hemoglobin level at POD1 was identified as s predictive factor of ADE in our study. This finding is supported by a previous study suggesting that drug-induced anemia is common with enoxaparin treatment. [29]. In addition, we observed that the hemoglobin value at POD7 was significantly lower than POD1 in all 13 anemia patients (median: 8.9 g/dL vs. 7.5 g/dL; Additional file 3: Figure S1). No significant relationship was observed between bleeding and anemia in the patients as a whole (Additional file 4: Table S3). Furthermore, the volume of bleeding during surgery was not correlated with the hemoglobin value at POD7 (Additional file 5: Figure S2). Although the precise molecular mechanisms are still unknown, our results suggest that enoxaparin has the potential to decrease hemoglobin levels and induce anemia. Enoxaparin may increase serum alkaline phosphatase (ALP) and lactate dehydrogenase (LDH) levels after orthopedic operation with high rate (Clexane® [interview form]. KAKEN PHARMACEUTICAL CO., LTD., Japan; Mar 2015, http://www.kaken.co.jp/medical/if/clexane_201503if.pdf, Accessed Dec 20, 2016). In this study, we also observed the increase in serum ALP and LDH levels after (POD7) the treatment of enoxaparin compared with before treatment (POD1) (Additional file 6: Figure S3). Although the degrees of the increase in these parameters did not meet the criteria for ADEs in this study, serum ALP and LDH levels should be carefully monitored.

This study has some limitations. First, we did not evaluate the serum albumin level and non-bound enoxaparin concentration. Enoxaparin seems to mainly bind to albumin. Albumin constitutes > 50% of serum total protein and serum albumin level is generally relative to serum total protein level. However, our data lacked information about serum albumin levels, so we could not completely clarify a potential mechanism for serum total protein affecting the effectiveness and safety of enoxaparin. As discussed above, hypoproteinemia seems to be independently associated with the induction of DVT and indirectly affects the efficacy of enoxaparin [28]. Further studies will be needed to identify the pharmacokinetics and pharmacodynamics of enoxaparin in patients with and without hypoalbuminemia. Second, the population in this study has gender bias because the prevalence of osteoarthritis-related disability is higher among women than men [30, 31]. In this study, we were not able to indicate clearly any gender differences due to the small number of male patients compared with female patients. Because most TKA patients are female and the validity of our findings in male TKA patients is unclear, further large scale studies will be needed to evaluate the gender differences. Third, we did not conduct a VTE risk evaluation in this study. Although the information about DVT risk criteria (such as Wells score for DVT) is critical for evaluation of the efficacy of enoxaparin, we lacked the parameters needed to calculate Wells score in this study. Fourth, several factors (such as volume of bleeding during surgery, ALT, γ-GTP, CRP, and concomitant drugs) were below the necessary limit of power (i.e. 80%) to predict the effectiveness and/or ADE of enoxaparin. Therefore, further investigations with a larger sample size are required before any definitive conclusions can be made. Finally, in this study, elevation of liver function tests, such as AST, ALT and γ-GTP, was observed as an ADE in 4 patients (Additional file 1: Table S1). We also conducted a preliminary analysis of the relationship between the abnormality in the liver function tests and hemoglobin levels. However, significant correlations were not observed among these parameters (Additional file 7: Figures S4 and Additional file 8 Figure S5). In the multivariate logistic regression analysis, we identified serum hemoglobin as a factor associated with ADE of enoxaparin in aged patients. However, the abnormalities of serum liver parameters might not be related to serum hemoglobin. Any possible link between lower blood hemoglobin at POD1 and the higher incidence of total ADEs may be masked by the incidence of anemia (identified by blood hemoglobin levels at POD7). In this study we did not clearly evaluate the relationship between blood hemoglobin and other ADEs, such as abnormality of liver function and bleeding because of the insufficient power of the analysis. Further study to clarify the relationship between blood hemoglobin and other ADEs will also be needed.

## Conclusions

In this study, we have identified candidate factors, such as serum total protein and hemoglobin levels, related to the effectiveness and safety of enoxaparin treatment for TKA in aged Japanese patients. The results suggest that serum total protein level before treatment of enoxaparin relates to the effectiveness and safety of enoxaparin in a Japanese aged population. In addition, the results indicate that patients should be carefully monitored for development of anemia during enoxaparin treatment, particularly those with lower levels of serum hemoglobin before treatment. Further large scale studies are needed to clarify the factors described.

## Additional files

Additional file 1: Table S1. Comparison of the incidence rate of adverse drug events (ADE) in the effective and ineffective groups. (DOC 70 kb)
Additional file 2: Table S2. Classification of the adverse drug events reported in this study Adverse drug events. (DOC 71 kb)
Additional file 3: Figure S1. Hemoglobin levels at POD1 and POD7 in anemia patients. In all 13 patients in the anemia (+) group, the hemoglobin value at POD 7 was lower than POD 1 with a significant difference (median: 8.9 g/dL vs 7.5 g/dL). The bottom and top of the box show the 25 and 75% rankings and therefore the interquartile range. The minimum and maximum rankings are denoted by the lower and upper whiskers. Outliers are denoted by circles. The two groups were compared using Wilcoxon signed-rank test. (PPTX 65 kb)
Additional file 4: Table S3. Comparison of the incidence rate of anemia in the bleeding (+) group and bleeding (-) group (DOC 70 kb)
Additional file 5: Figure S2. Correlation between volume of bleeding during surgery and hemoglobin at post-operative day 7 (POD7). Significant correlation was not observed between volume of bleeding during surgery and hemoglobin at POD7. Statistical analysis were performed using Pearson correlation coefficient. The red ellipse represents 95% confidence interval. (PPTX 65 kb)
Additional file 6: Figure S3. Clinical laboratory data at post-operative day (POD) 1 and POD7Alkaline phosphatase (ALP) and lactate dehydrogenase (LDH) values at POD7 are significantly higher than POD1 (ALP (median): 185 IU/L vs 208 IU/L, LDH (median): 200 IU/L vs 214 IU/L, respectively). The bottom and top of the box show the 25 and 75% rankings and therefore the interquartile range. The minimum and maximum rankings are denoted by the lower and upper whiskers. Outliers are denoted by circles. Two groups were compared by Wilcoxon signed-rank. (PPTX 86 kb)
Additional file 7: Figure S4. Correlation between liver function test value post-operative day (POD) 7 and hemoglobin at POD7. Significant correlation was not observed between hemoglobin level at POD7 and liver function tests POD7, such as aspartate aminotransferase, alanine aminotransferase and gamma-glutamyl transferase. Statistical analysis were performed using Pearson correlation coefficient. The red ellipse represents 95% confidence interval. (PPT 182 kb)
Additional file 8: Figure S5. Correlation between liver function test value at post-operative day (POD) 7 and hemoglobin at POD1. Significant correlation was not observed between hemoglobin level POD1 and liver function tests POD7, such as aspartate aminotransferase, alanine aminotransferase and gamma-glutamyl transferase. Statistical analysis were performed using Pearson correlation coefficient. The red ellipse represents 95% confidence interval. (PPT 185 kb)

## Abbreviations

ADE: Adverse drug events
BIC: Bayesian information criterion
CRNM: Clinically relevant non-major
DVT: Deep vein thrombosis
POD1: Postoperative day 1
ROC: Receiver operating characteristic
TKA: Total knee arthroplasty
VTE: Venous thromboembolism
