# Supplementary material for: Predictive factors for effectiveness and safety of enoxaparin for total knee arthroplasty in aged Japanese patients: a retrospective review
Source: J Pharm Health Care Sci. 2017 Jan 18;3:6. doi: 10.1186/s40780-017-0075-x (PMC5241995; doi:10.1186/s40780-017-0075-x)
Supplement: Additional file 1: Table S1. — Comparison of the incidence rate of adverse drug events (ADE) in the effective and ineffective groups. (DOC 70 kb) [file 40780_2017_75_MOESM1_ESM.doc]

**Supplemental Table 1**

**Comparison of the incidence rate of adverse drug events (ADE) in the effective and ineffective groups**

|  | **ADE (-)** | **ADE (+)** | *P* value |
| --- | --- | --- | --- |
| Efficacy, no. (%)  effective  ineffective | 89 (82.4) 19 (17.6) | 16 (80.0)  4 (20.0) | 0.76 |

The two groups were compared using Fisher's exact test. The data indicated no significant difference between the groups.
