# Supplementary material for: Predictive factors for effectiveness and safety of enoxaparin for total knee arthroplasty in aged Japanese patients: a retrospective review
Source: J Pharm Health Care Sci. 2017 Jan 18;3:6. doi: 10.1186/s40780-017-0075-x (PMC5241995; doi:10.1186/s40780-017-0075-x)
Supplement: Additional file 2: Table S2. — Classification of the adverse drug events reported in this study Adverse drug events. (DOC 71 kb) [file 40780_2017_75_MOESM2_ESM.doc]

**Supplemental Table 2 Classification of the adverse drug events reported in this study**

| **Adverse drug events** | **Number (%)** |
| --- | --- |
| Anemia | 13 (65.0) |
| liver function abnormalities | 4 (20.0) |
| CRNM bleeding | 4 (20.0) |
| urticaria | 1 (5.0) |

*CRNM* indicates clinically relevant non-major.
