# Supplementary material for: Predictive factors for effectiveness and safety of enoxaparin for total knee arthroplasty in aged Japanese patients: a retrospective review
Source: J Pharm Health Care Sci. 2017 Jan 18;3:6. doi: 10.1186/s40780-017-0075-x (PMC5241995; doi:10.1186/s40780-017-0075-x)
Supplement: Additional file 3: Figure S1. — Hemoglobin levels at POD1 and POD7 in anemia patients. In all 13 patients in the anemia (+) group, the hemoglobin value at POD 7 was lower than POD 1 with a significant difference (median: 8.9 g/dL vs 7.5 g/dL). The bottom and top of the box show the 25 and 75% rankings and therefore the interquartile range. The minimum and maximum rankings are denoted by the lower and upper whiskers. Outliers are denoted by circles. The two groups were compared using Wilcoxon signed-rank test. (PPTX 65 kb) [file 40780_2017_75_MOESM3_ESM.pptx]

## Slide 1
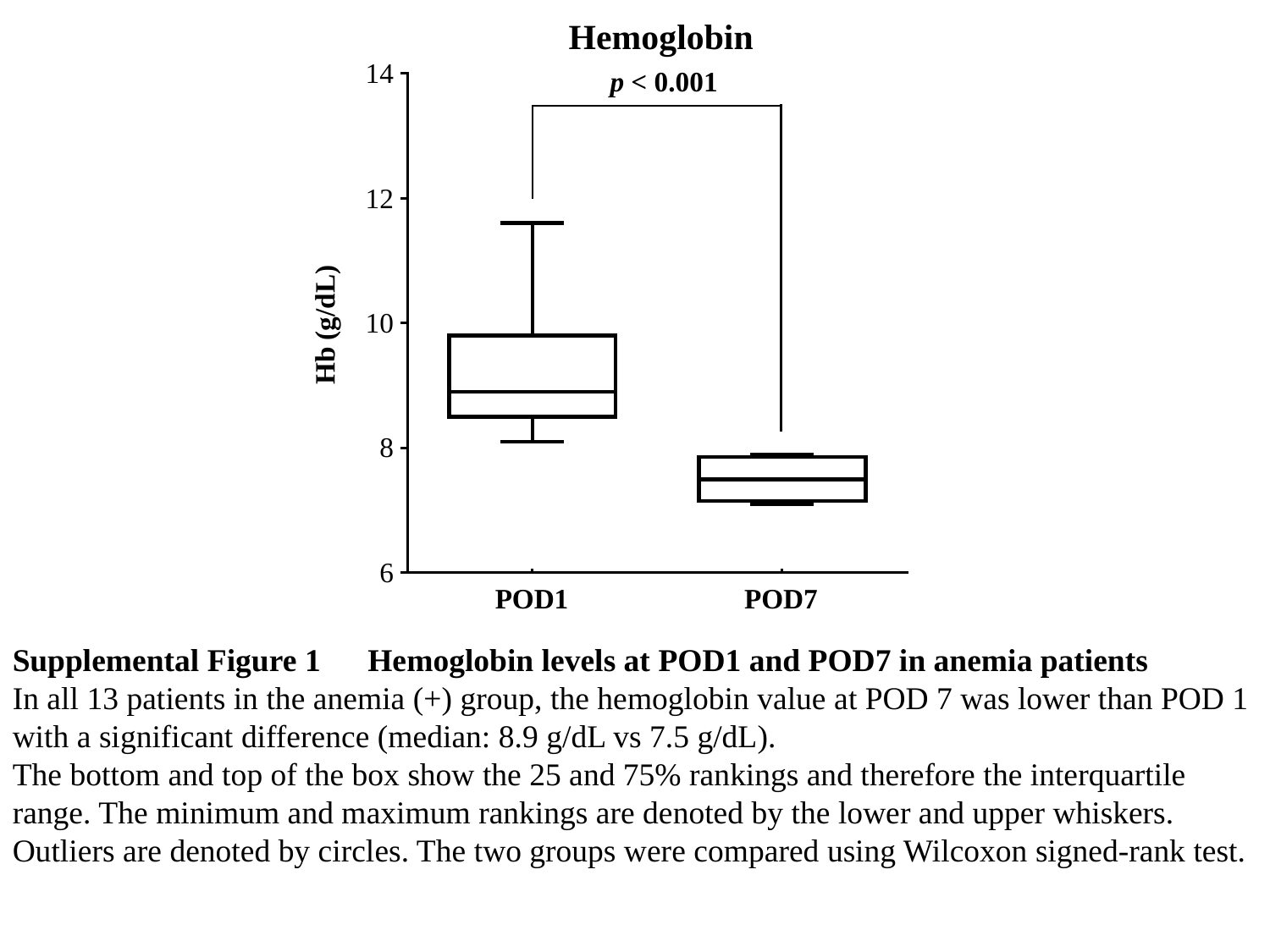

Hemoglobin
14
p < 0.001
12
10
Hb (g/dL)
8
6
POD7
POD1
Supplemental Figure 1　Hemoglobin levels at POD1 and POD7 in anemia patients
In all 13 patients in the anemia (+) group, the hemoglobin value at POD 7 was lower than POD 1 with a significant difference (median: 8.9 g/dL vs 7.5 g/dL).
The bottom and top of the box show the 25 and 75% rankings and therefore the interquartile range. The minimum and maximum rankings are denoted by the lower and upper whiskers. Outliers are denoted by circles. The two groups were compared using Wilcoxon signed-rank test.
