# Supplementary material for: Predictive factors for effectiveness and safety of enoxaparin for total knee arthroplasty in aged Japanese patients: a retrospective review
Source: J Pharm Health Care Sci. 2017 Jan 18;3:6. doi: 10.1186/s40780-017-0075-x (PMC5241995; doi:10.1186/s40780-017-0075-x)
Supplement: Additional file 4: Table S3. — Comparison of the incidence rate of anemia in the bleeding (+) group and bleeding (-) group (DOC 70 kb) [file 40780_2017_75_MOESM4_ESM.doc]

**Supplemental Table 3**

**Comparison of the incidence rate of anemia in the bleeding (+) group and bleeding (-) group**

|  | **Anemia (-)** | **Anemia (+)** | *P* value |
| --- | --- | --- | --- |
| Bleeding, no. (%)  Bleeding (-)  Bleeding (+) | 112 (97.4)  3 (2.6) | 12 (92.3)  1 (7.7) | 0.352 |

The two groups were compared using Fisher's exact test. The data indicated no significant difference between the groups.
