# Supplementary material for: Predictive factors for effectiveness and safety of enoxaparin for total knee arthroplasty in aged Japanese patients: a retrospective review
Source: J Pharm Health Care Sci. 2017 Jan 18;3:6. doi: 10.1186/s40780-017-0075-x (PMC5241995; doi:10.1186/s40780-017-0075-x)
Supplement: Additional file 5: Figure S2. — Correlation between volume of bleeding during surgery and hemoglobin at post-operative day 7 (POD7). Significant correlation was not observed between volume of bleeding during surgery and hemoglobin at POD7. Statistical analysis were performed using Pearson correlation coefficient. The red ellipse represents 95% confidence interval. (PPTX 65 kb) [file 40780_2017_75_MOESM5_ESM.pptx]

## Slide 1
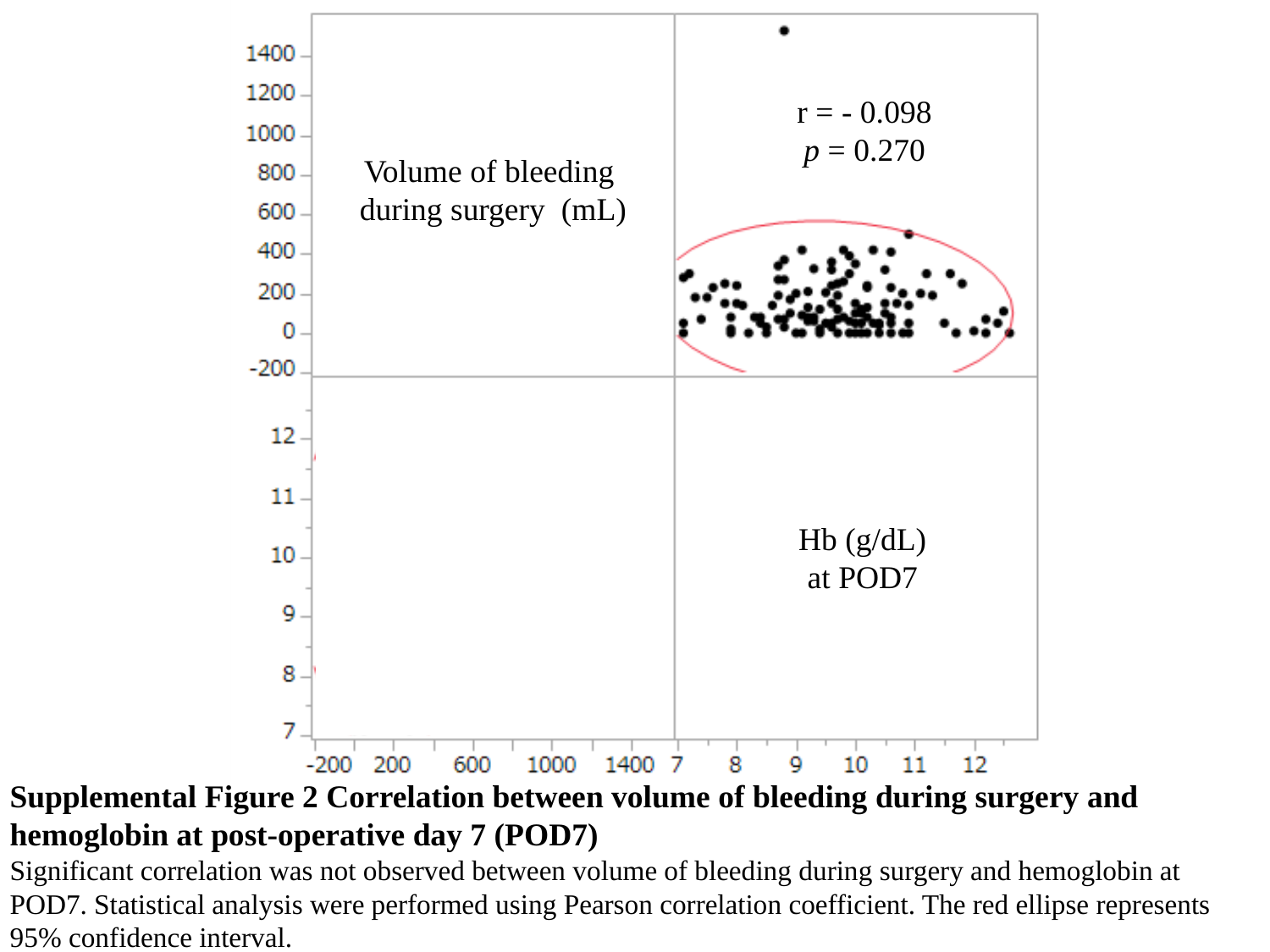

r = - 0.098
p = 0.270
Volume of bleeding
during surgery (mL)
Hb (g/dL)
at POD7
Supplemental Figure 2 Correlation between volume of bleeding during surgery and hemoglobin at post-operative day 7 (POD7)
Significant correlation was not observed between volume of bleeding during surgery and hemoglobin at POD7. Statistical analysis were performed using Pearson correlation coefficient. The red ellipse represents 95% confidence interval.
