# Supplementary material for: Predictive factors for effectiveness and safety of enoxaparin for total knee arthroplasty in aged Japanese patients: a retrospective review
Source: J Pharm Health Care Sci. 2017 Jan 18;3:6. doi: 10.1186/s40780-017-0075-x (PMC5241995; doi:10.1186/s40780-017-0075-x)
Supplement: Additional file 6: Figure S3. — Clinical laboratory data at post-operative day (POD) 1 and POD7Alkaline phosphatase (ALP) and lactate dehydrogenase (LDH) values at POD7 are significantly higher than POD1 (ALP (median): 185 IU/L vs 208 IU/L, LDH (median): 200 IU/L vs 214 IU/L, respectively). The bottom and top of the box show the 25 and 75% rankings and therefore the interquartile range. The minimum and maximum rankings are denoted by the lower and upper whiskers. Outliers are denoted by circles. Two groups were compared by Wilcoxon signed-rank. (PPTX 86 kb) [file 40780_2017_75_MOESM6_ESM.pptx]

## Slide 1
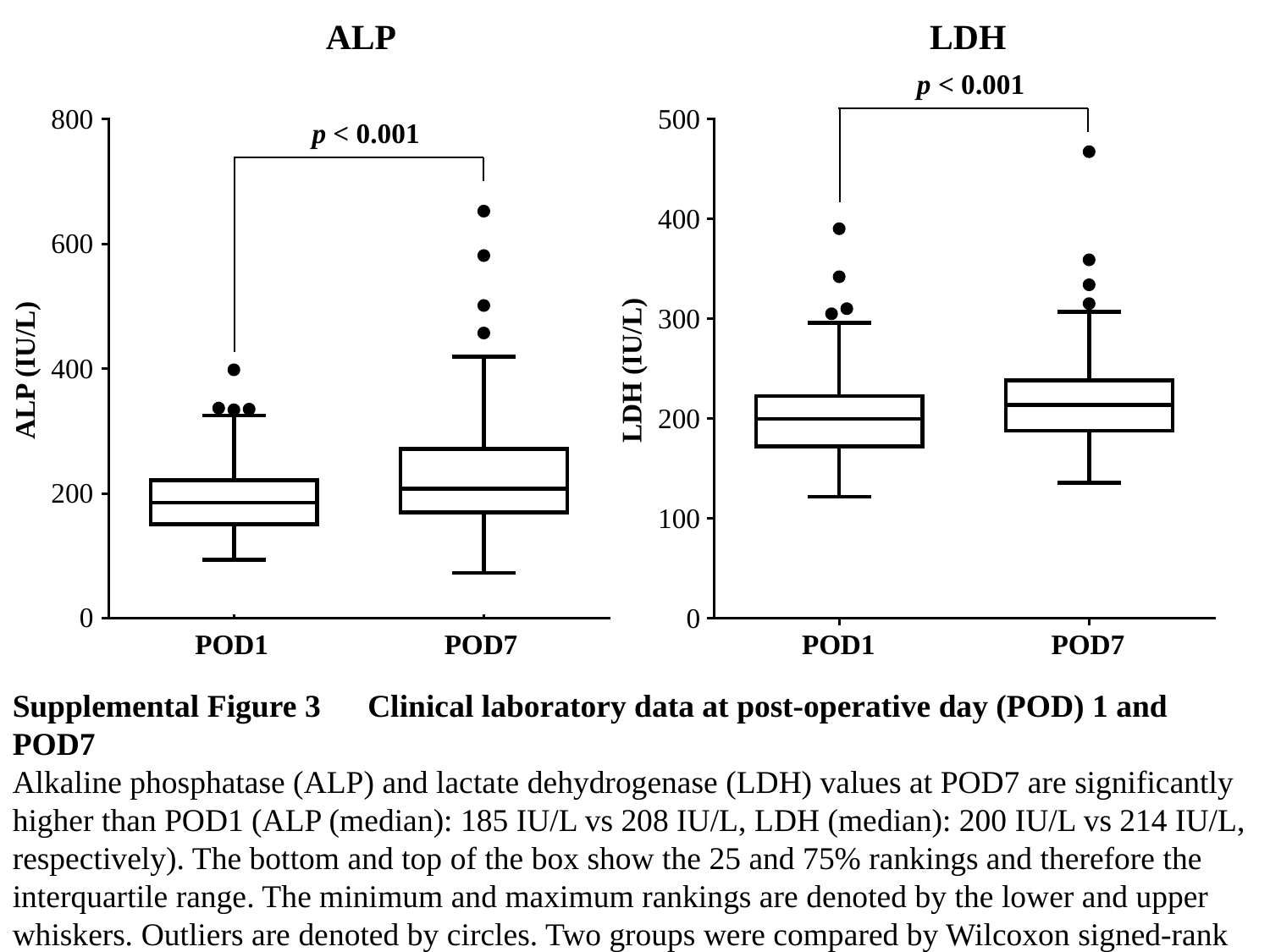

ALP
800
p < 0.001
600
400
ALP (IU/L)
200
0
POD7
POD1
LDH
p < 0.001
500
400
300
LDH (IU/L)
200
100
0
POD7
POD1
Supplemental Figure 3　Clinical laboratory data at post-operative day (POD) 1 and POD7
Alkaline phosphatase (ALP) and lactate dehydrogenase (LDH) values at POD7 are significantly higher than POD1 (ALP (median): 185 IU/L vs 208 IU/L, LDH (median): 200 IU/L vs 214 IU/L, respectively). The bottom and top of the box show the 25 and 75% rankings and therefore the interquartile range. The minimum and maximum rankings are denoted by the lower and upper whiskers. Outliers are denoted by circles. Two groups were compared by Wilcoxon signed-rank test.
