# Supplementary material for: Predictive factors for effectiveness and safety of enoxaparin for total knee arthroplasty in aged Japanese patients: a retrospective review
Source: J Pharm Health Care Sci. 2017 Jan 18;3:6. doi: 10.1186/s40780-017-0075-x (PMC5241995; doi:10.1186/s40780-017-0075-x)
Supplement: Additional file 8: Figure S5. — Correlation between liver function test value at post-operative day (POD) 7 and hemoglobin at POD1. Significant correlation was not observed between hemoglobin level POD1 and liver function tests POD7, such as aspartate aminotransferase, alanine aminotransferase and gamma-glutamyl transferase. Statistical analysis were performed using Pearson correlation coefficient. The red ellipse represents 95% confidence interval. (PPT 185 kb) [file 40780_2017_75_MOESM8_ESM.ppt]

## Slide 1
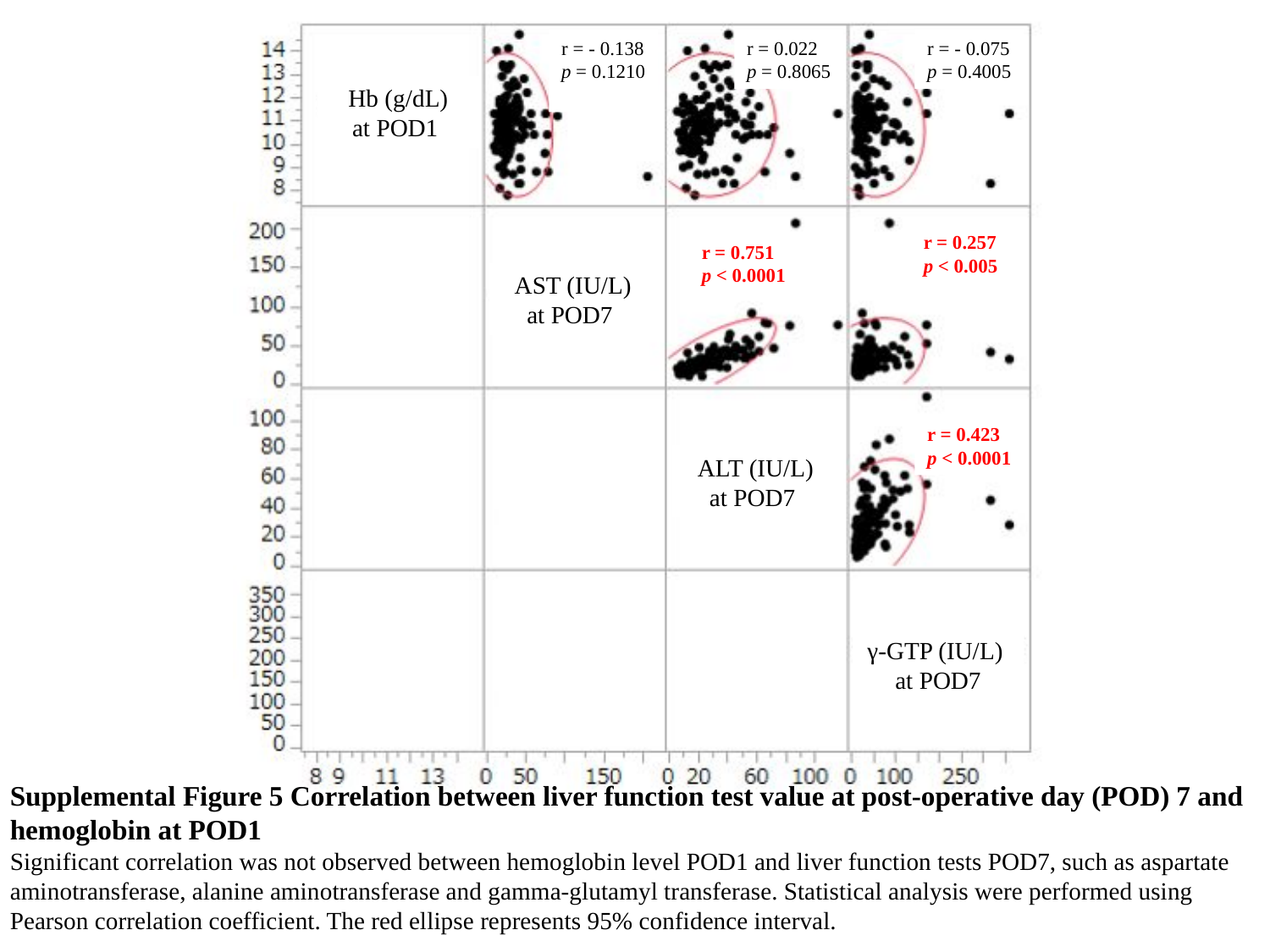

r = - 0.138
p = 0.1210
r = 0.022
p = 0.8065
r = - 0.075
p = 0.4005
Hb (g/dL)
at POD1
r = 0.257
p < 0.005
r = 0.751
p < 0.0001
AST (IU/L)
at POD7
r = 0.423
p < 0.0001
ALT (IU/L)
at POD7
γ-GTP (IU/L)
at POD7
Supplemental Figure 5 Correlation between liver function test value at post-operative day (POD) 7 and hemoglobin at POD1
Significant correlation was not observed between hemoglobin level POD1 and liver function tests POD7, such as aspartate aminotransferase, alanine aminotransferase and gamma-glutamyl transferase. Statistical analysis were performed using Pearson correlation coefficient. The red ellipse represents 95% confidence interval.
